# Evidence for a bimodal distribution of hybrid indices in a hybrid zone with high admixture

**DOI:** 10.1098/rsos.150285

**Published:** 2015-12-09

**Authors:** Jessica L. McKenzie, Rashpal S. Dhillon, Patricia M. Schulte

**Affiliations:** 1 Department of Zoology, University of British Columbia, Vancouver, British Columbia, Canada; 2 Centre for Aquaculture and Environmental Research, Fisheries and Oceans Canada, West Vancouver, British Columbia, Canada

**Keywords:** fish, hybrid zone, bimodal, mtDNA, hybrid index, reproductive isolation

## Abstract

The genetic structure of a hybrid zone can provide insights into the relative roles of the various factors that maintain the zone. Here, we use a multilocus approach to characterize a hybrid zone between two subspecies of killifish (*Fundulus heteroclitus*, Walbaum 1792) found along the Atlantic coast of North America. We first analysed clinal variation along the Atlantic coast using a single-nucleotide polymorphism in the mitochondrial DNA (mtDNA) displacement loop (D-loop) and a panel of nine nuclear microsatellite markers. A model constraining all clines to the same width and centre was not significantly different from a model in which the clines were allowed to vary independently. Locus-by-locus analysis indicated that the majority of nuclear clines shared the same centre as the mtDNA cline, and the widths of these clines were also narrower than that predicted by a neutral model, suggesting that selection is operating to maintain the hybrid zone. However, two of the nuclear clines had widths greater than the neutral prediction and had centres that were displaced relative to the mtDNA cline centre. We also found that a marsh located near the centre of the mtDNA cline demonstrated a bimodal distribution of nuclear hybrid index values, suggesting a deficit of first-generation hybrids and backcrossed genotypes. Thus, selection against hybrid genotypes may be playing a role in maintaining this hybrid zone and the associated steep nuclear and mtDNA clines.

## 1. Introduction

When two recently diverged taxa meet and reproductive isolation is not complete, hybridization can occur, which causes a transition between the morphological, physiological and ultimately genetic traits of the two taxa as one crosses the hybrid zone [1,2]. Hybrid zones, and the resulting phenotypic and genetic clines that they create [1,3,4], provide natural laboratories for the study of speciation and natural selection [2] and can provide insight into the genes underlying morphological, physiological and reproductive differences among taxa (e.g. [5]).

Patterns of genetic variation within a hybrid zone can help illuminate the factors that maintain the zone [6–8]. For example, analysis of the shape of clines can be used to infer the strength of selection on various regions of the genome [9], because steep clines with cline widths narrower than predicted by a neutral model suggest that the differentiated alleles at these loci (or closely linked loci) are being maintained by selection [10,11]. Another approach to the analysis of hybrid zones involves explicit consideration of multilocus genetic variation through the calculation of the hybrid index, which is an estimate of the similarity of each hybrid individual to the parental types. By examining the frequency distribution of hybrid indices, it is possible to assess the extent of reproductive isolation operating within the zone [7]. If there is limited reproductive isolation between the hybridizing taxa, a unimodal pattern of hybrid indices will be observed at the centre of the zone, indicative of a hybrid swarm, whereas if there is substantial reproductive isolation between the taxa, a bimodal pattern of hybrid indices will be observed [7]. Observations of Hardy–Weinberg equilibrium (HWE) and linkage equilibrium at multiple loci are typical in unimodal zones, whereas the presence of heterozygote deficit and linkage disequilibrium (and cytonuclear disequilibrium) suggests that reproductive isolation and/or local adaptation of either parental type to divergent habitats are acting to reduce the success of hybrid offspring [7].

It is important to recognize that within a given hybrid zone different loci may show differing degrees of bimodality, depending on the strength of selection acting on a particular locus [7]. Similarly, the locations of the cline centres and steepness of clines may vary among loci, with some markers yielding steep, abrupt clines and others more gradual cline shapes, depending on the involvement or linkage of these markers to traits involved in reproductive isolation [12,13]. Thus, determining the pattern of genetic variation across and within a hybrid zone is an important step in identifying the factors that are responsible for the maintenance of the zone.

Here, we examine the patterns of genetic variation in a hybrid zone between two subspecies of Atlantic killifish, *Fundulus heteroclitus*. Morphological, physiological and genetic clines in *F. heteroclitus* have been extensively studied, and this species is cited as a ‘textbook example’ of intraspecific latitudinal genetic variation (e.g. [14,15]). Previous biochemical genetic studies in killifish have identified clines along the Atlantic coast at various enzyme-coding loci, with northern alleles being replaced by southern alleles along the New Jersey shoreline [16,17]. Subsequent work at the molecular level has detected clines in mitochondrial DNA (mtDNA) [18,19], putatively neutral nuclear microsatellite loci [20,21] and a variety of nuclear single-nucleotide polymorphisms (SNPs) [22]. Most of these genetic and character clines show breaks from one predominant type to the other between 40° N and 41° N, which has been used as evidence to suggest that *F. heteroclitus* consists of two subspecies: *Fundulus heteroclitus heteroclitus* Linnaeus 1766 (hereafter referred to as ‘the southern subspecies’) ranging from New Jersey south to northern Florida and *Fundulus heteroclitus macrolepidotus* Walbaum 1792 (hereafter referred to as ‘the northern subspecies’) ranging from Connecticut north to Newfoundland [23], with a hybrid zone located along the coast of New Jersey connecting the two subspecies.

For example, there has been thorough sampling of the area comprising the suspected transition zone and analysis using restriction fragment site polymorphisms (RFLPs) in the mitochondrial genome [19], and these authors concluded that the zone of intergradation occurred in a 60 km stretch between Barnegat Light, New Jersey (39.77° N, 74.12° W) and Great Egg, New Jersey (39.30° N, 74.63° W). Interestingly, in a more recent study employing nuclear markers [20], data from eight microsatellite loci were used to estimate q-bar (structure) [24,25] leading to the conclusion that the centre of the hybrid zone was located approximately at Newark Bay, New Jersey (40.69° N, 74.11° W), roughly 130 km north of the most northern limit described for the mitochondrial inflection point. Despite the extensive work that has been conducted in this system, no previous study has simultaneously examined patterns of variation in both the nuclear and mitochondrial genomes in the putative zone of contact, nor have multilocus approaches been applied.

Here, we aimed to increase the sampling intensity in the putative hybrid zone, focusing on sampling from marshes located close to the mitochondrial cline centre, as the mtDNA cline is the steepest that has yet been detected in this species [22]. We used a combination of mtDNA and nine microsatellite markers to examine populations of killifish from marshes located within and immediately surrounding this region, conducting clinal analyses and assessing coincidence and concordance of these clines. We then used the microsatellite data to calculate multilocus hybrid indices for all individuals sampled to determine the pattern of the genetic variation within the hybrid zone with respect to the unimodal–bimodal continuum, to provide additional insight into the nature of this hybrid zone.

## 2. Material and methods

### 2.1 Fish collection

**Figure 1.**
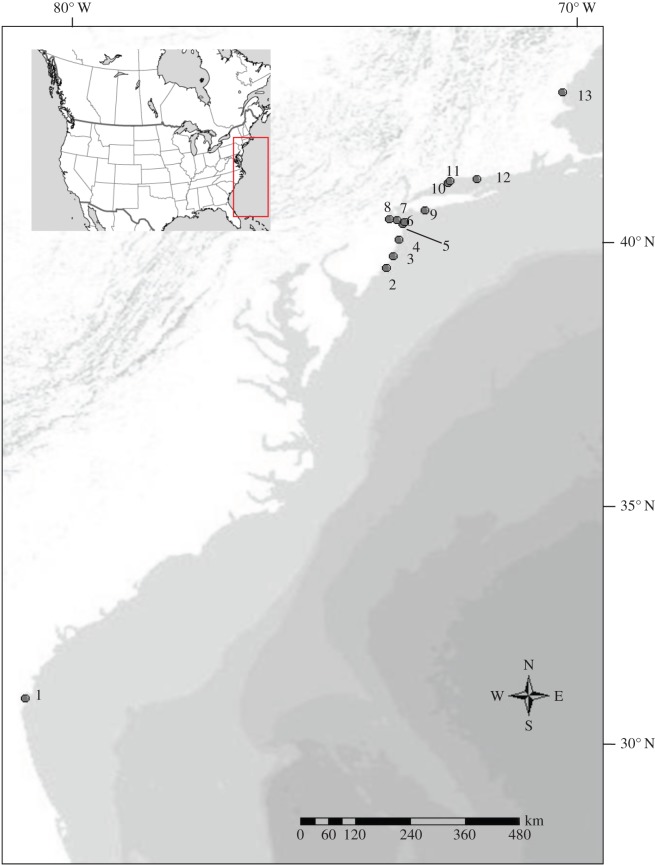
Map of collection locations. Numbers correspond to those listed in table 1. Inset of North America indicates location of sampling area.

*Fundulus heteroclitus* (520 individuals) were collected from various locations along the Atlantic coast of North America, with sample collection concentrated in and immediately adjacent to the presumed hybrid zone between the two subspecies in New Jersey (table 1 and figure 1). Samples from the contact zone were collected from May to June of 2008 from 11 locations along the coast (at the mouth of each marsh). Minnow traps (G-type) were used and trap set time ranged from 2 to 6 h. Fish length was recorded and a fin clip was taken from each individual and preserved in 95% ethanol. Additional samples representative of the pure populations of the two subspecies from previous studies [26] were collected near Brunswick, GA and Hampton, NH (table 1).

**Table 1.** Sampling location names, coordinates and sample sizes (*n*) along the Atlantic coast of North America.

location name	distance from Georgia (km)	latitude (°N)	longitude (°W)	*n*
1. Brunswick, GA	0	31.116130	−81.462250	19
2. Rutgers University Marine Field Station (RUMFS), NJ	1145.77	39.512905	−74.320450	50
3. Laurel Harbour, NJ	1186.03	39.750349	−74.192734	50
4. Metedeconk Creek, NJ	1208.94	40.065616	−74.065320	49
5. Navesink River, NJ	1228.41	40.376297	−73.993993	13
6. Sandy Hook Bay, NJ	1231.88	40.412904	−73.969487	50
7. Belford Creek, NJ	1242.06	40.440968	−74.103963	49
8. Cheesequake Creek, NJ	1257.54	40.463420	−74.258888	50
9. Wantagh, NY	1285.31	40.638689	−73.563080	39
10. Housatonic River, CT	1357.23	41.173856	−73.103081	22
11. Roger’s Creek, CT	1363.41	41.213379	−73.057564	12
12. Hammonassett Creek, CT	1393.09	41.251971	−72.537634	48
13. Hampton, NH	1631.00	42.937055	−70.839844	20

### 2.2 Mitochondrial D-loop PCR and SNP genotyping

DNA was extracted from all samples using Qiagen’s DNeasy® Blood and Tissue Kit and all polymerase chain reactions were performed using an MJ Research PTC-200 Peltier Thermal Cycler. A restriction digestion assay was developed to allow the rapid diagnosis of mitochondrial-type based on a single fixed difference found during a preliminary screening of northern (*n*=20) and southern (*n*=20) mtDNA D-loop sequences. Preliminary screening was performed using primers K (forward) and G (reverse) [27] to amplify approximately 1100 base pairs (bp) of the mtDNA D-loop and reactions were prepared in 12.5 μl volumes containing 10× Taq Buffer with KCl (Fermentas), 2.0 mM MgCl_2_, 0.2 mM dNTPs, 0.4 μM each primer, 1 unit Taq DNA polymerase (Fermentas), the appropriate volume of ddH_2_O and 1.0 μl genomic DNA of varying concentrations. The amplification profile consisted of an initial denaturing step of 2 min at 95°C followed by five cycles of denaturation at 94.0°C for 30 s, primer annealing at 60°C for 45 s, extension at 72°C for 1 min 30 s, and 34 cycles of denaturation at 94°C for 30 s, primer annealing at 65°C for 45 s, extension at 72°C for 1 min 30 s. The final extension was at 72°C for 5 min. The resulting PCR products were then electrophoresed on a 1% agarose gel pre-stained with ethidium bromide and visualized using a GeneGenius Bio Imaging System (Syngene). Successful amplifications were purified using Qiagen’s QIAquick® PCR Purification Kit and direct sequenced using the BigDye^TM^ Terminator Cycle Sequencing Ready Reaction Kit (Perkin-Elmer Corporation, Foster City, CA, USA). This reaction contained 3.0 μl BigDye® dilution mix, 2.0 μl purified PCR product, 1.0 μM of K primer and ddH_2_O for a final volume of 10 μl. The cycle sequencing profile consisted of an initial denaturing step of 96°C for 1 min followed by 26 cycles of 96°C for 10 s, 50°C for 5 s and 60°C for 4 min. The sequences were edited and aligned using the program MEGA 4.0.2 [28]. The online program NEBcutter v.2.0 [29] was then used to identify a restriction enzyme that would cut at one of the several diagnostic sites that exhibited fixed differences between northern and southern fish.

The restriction digestion assay was performed by PCR, amplifying the mtDNA D-loop as above. 8.0 μl reactions containing 5 μl of PCR product, 10× Buffer ScaI (Fermentas), 0.25 units of the restriction enzyme *Sca*I (Fermentas) and the appropriate volume of ddH_2_O were incubated for 3 h at 37°C. The undigested 1100 bp fragment represented individuals with a northern mitochondrial haplotype while the digested 900 bp fragment was diagnostic of the southern mitotype. This PCR-RFLP assay was then applied to all 520 fish collected from 13 locations (table 1). The fragment polymorphisms generated by the restriction digest were easily discernible on a 1.5% agarose gel pre-stained with ethidium bromide. Ten northern, nine southern and 28 hybrid zone fish were then sequenced at random to confirm the results of the RFLP assay. The resulting sequences have been submitted to GenBank (Accession Numbers JQ518219–JQ518265).

### 2.3 Microsatellite genotyping

Individuals from locations 1 to 13 inclusive were also genotyped at nine microsatellite loci [30]: FhCA-1, FhATGB101, FhATGB128, FhATG-2, FhATG-4, FhATG-6, FhATG-17, FhATG-18 and FhATG-20. PCR conditions for each of these microsatellite markers required modification from those described by Adams *et al*. [30] for successful amplification (electronic supplementary material, table S1). Reactions involving a single round of denaturation, annealing and extension were performed for 35 cycles. For any reaction involving two rounds of denaturation, annealing and extension, the first round proceeded for five cycles, followed by a second round of 30 cycles. All final extensions were performed at 72° for 5 min. FhATG-17, FhATG-18 and FhATG-20 were combined in a single multiplex reaction, as were FhATG-2 and FhATG-4. Although FhATGB101 and FhATGB-128 used identical reaction conditions as FhATG-2 and FhATG-4, they were combined in a separate multiplex reaction to avoid complications as a result of size-fragment overlap. Final reaction volumes were 10 μl with reagent concentrations as follows: 10× Taq Buffer with KCl (Fermentas), 2.0 mM MgCl_2_, 0.2 mM dNTPs, 0.4 μM LDH-F1, 0.4 μM LDH-R2, 0.5 units Taq DNA polymerase (Fermentas), 1.0 μl genomic DNA and ddH_2_O for a final reaction volume of 10 μl. All reactions were performed with these concentrations except for the multiplex reaction involving FhATG-2 and FhATG-4 which required 1 unit of Taq polymerase. PCR products were then dried down in 96-well plates and sent to the Boston Children’s Hospital Molecular Genetics Core Facility for genotyping on an Applied Biosystems 3730 DNA Analyzer. Alleles were scored using Peak Scanner^TM^ software v. 1.0 (Applied Biosystems). Multilocus microsatellite genotypes for all individuals used in this study can be found in the electronic supplementary material, table S2.

### 2.4 Analyses

Microchecker v. 2.2.3 [31] was used to screen the microsatellite data for the presence of null alleles, large allele dropout and scoring errors. The program Genepop v. 4.0.10 [32] was used to conduct the exact test for Hardy–Weinberg equilibrium. Tests for heterozygote excess and heterozygote deficiency were performed for each locus in each population. Markov chain parameters were set to the defaults (dememorization number=1000; 100 batches; 1000 iterations per batch). Genetix v. 4.05.2 [33] was used to test for linkage disequilibrium among the different microsatellite loci. Each locus pair in each population was tested for evidence of linkage disequilibrium using the Black and Krafsur procedure [34]. The program CNDm (available at http://statgen.ncsu.edu/cnd/CNDd.php [35,36]) was used to test for cytonuclear disequilibrium between mitochondrial-type and each microsatellite locus within marshes 2–8 (table 1).

### 2.5 Clinal analyses

The program HZAR (hybrid zone analysis for R) [37], implemented in the R programming environment [38], was used to fit all clines and perform all clinal analyses. First, the best of three models (as determined through lowest AIC value) was fit to the allele frequency data and used to predict cline parameters (centre, width, *p*_min_,*p*_max_). In addition to the null model, the models used for the cline fitting of the molecular markers were: model I: fixed *p*_min_/*p*_max_ values and tails not fitted, model II: *p*_min_/*p*_max_ estimated and tails not fitted, and model III: *p*_min_/*p*_max_ estimated and tails fitted. More detailed descriptions of these models can be found in Brumfield *et al.* [39]. Once the best model had been selected, it was used to plot the best fit cline for the observed allele frequencies as well as the 2-unit support envelope around the cline. In order to compare the estimate for cline widths to that which would be expected under a neutral model of selection (*w*), the following equation [40], was solved for *w*:

2.1 $$T={\left(\frac{w}{(\sqrt{2\pi })\sigma }\right)}^{2},$$

where *T* represents the number of generations since contact under the assumption of a neutral cline, and *σ* represents dispersal per generation.

Clines were tested for coincidence and concordance using the program Cfit7 [41]. Briefly, this program uses the scaled logit shape to fit the clines [42]. Two models were compared: one in which mitochondrial and microsatellite clines were constrained to a common centre and width and one in which the cline shapes were allowed to vary independently. The likelihoods of these two models were then compared using the likelihood ratio test [43].

### 2.6 Hybrid index

The program structure 2.3.4 [24,25] was used to first identify the number of admixed populations among the 13 locations sampled, and second to approximate which of the sampled locations best represented the edges of the hybrid zone. The program was run using the default parameters, beginning with a 50 000 iteration burn-in followed by 150 000 Markov chain Monte Carlo iterations. The number of genetic clusters (*k*) tested ranged from one to 10, with each analysis being replicated 10 times. The number of clusters that best described the genetic admixture in our samples was determined using the method of Evanno *et al*. [44] as implemented in the online program Harvester [45]. Values of *q* were then examined to identify the populations bounding the hybrid zone.

Populations were deemed to represent the edges of the hybrid zone based on a shift in *q*-values from those indicative of either parental type predominating (0.10>*q*>0.90) to an increase in values representing admixed individuals (0.10<*q*<0.90). Individuals from these border marshes were then used as parental types to train the program introgress as implemented in the R programming environment [38,46,47]. This program uses allele frequency differentials (*δ*) to calculate a hybrid index for each individual based on their multilocus microsatellite genotypes. Hybrid index values for each individual in each marsh were then binned in 0.10 increments and compiled in a histogram representing the microsatellite hybrid indices present in each marsh. The shape of the frequency distribution (either unimodal or non-unimodal) was then determined by Hartigan’s diptest [48,49] using the package diptest as implemented in the R programming environment [38].

## 3. Results

### 3.1 Hardy–Weinberg Equilibrium and linkage disequilibrium

Among the microsatellites, there was no evidence of null alleles or large allele dropout, but there was some evidence of significant departures from HWE (table 2). Significant heterozygote deficit was observed for at least one locus in each of marshes 2, 3, 6, 11–13 (table 2). Significant heterozygote excess was observed at three loci (FhATG18, FhATG17 and FhCA-1) in marsh 12 and one locus (FhCA-1) among individuals collected from marsh 4. However, only one of these values (heterozygote deficit at locus FhATGB101 in marsh 6) remained significant following an adjustment for false discovery rate (*FDR*=0.05) using the classical one-stage method [50].

**Table 2.** Deviations from HWE (*F*_IS_) for microsatellite loci among coastal populations of *F. heteroclitus.*Location numbers correspond to those given in table 1. Positive *F*_IS_ indicates heterozygote deficit while negative *F*_IS_ indicates heterozygote excess. *F*_IS_ values significant at *α*=0.05 in bold. The final column indicates the number of pairs of loci showing significant linkage disequilibrium. The number of loci remaining significant after FDR-adjustment is shown in parentheses.

	locus name									
location	FhATG18	FhATG20	FhATG17	FhATGB101	FhATGB128	FhATG2	FhATG4	FhATG6	FhATG-CA1	no. pairs of loci in linkage disequilibrium
1.	0.081	0.029	0.161	−0.057	−0.186	−0.049	−0.067	−0.041	−0.000	6 (0)
2.	**0.154**	0.030	0.196	0.018	0.000	−0.015	−0.055	−0.018	0.009	7 (2)
3.	0.091	−0.068	0.123	−0.025	−0.086	−0.055	**0.061**	−0.025	−**0.083**	7 (2)
4.	0.041	−0.057	0.123	0.068	0.134	0.038	−0.099	0.044	−**0.112**	5 (2)
5.	−0.206	−0.039	0.189	0.028	0.052	−0.375	0.219	−0.146	−0.143	2 (0)
6.	−0.055	0.019	0.103	**0.127**^a^	0.045	−0.005	0.050	−0.069	−0.050	11 (8)
7.	0.018	0.042	0.201	0.044	−0.019	0.217	−0.047	−0.053	0.007	2 (0)
8.	−**0.168**	0.064	−0.061	−0.055	0.066	0.100	−0.006	−0.079	−0.067	4 (2)
9.	−0.110	0.083	0.070	**0.254**	−0.0041	−0.226	−0.021	0.002	−0.055	7 (2)
10.	−0.176	−0.097	−0.301	0.091	−0.147	−0.050	0.216	−0.086	−0.314	3 (0)
11.	−0.127	−0.029	0.018	n.a.	−0.177	n.a.	0.160	**0.500**	−0.065	2 (0)
12.	−**0.211**	−**0.097**	−**0.235**	−0.025	0.029	**0.490**	−0.090	−0.029	−**0.147**	2 (0)
13.	0.101	−0.162	−0.106	−0.062	**0.360**	n.a.	0.060	−0.018	−0.087	3 (0)

^a^*F*_IS_ values significant after FDR-adjustment.

Among the 13 locations sampled, a total of 61 pairs of microsatellite loci were found to be in significant linkage disequilibrium prior to correction for multiple testing, with 18 pairs of linkage disequilibria remaining significant after FDR-adjustment for multiple comparisons (table 2). No microsatellite loci were found to be in significant disequilibrium with the mitochondrial genome at any location.

## 4. Characterization of the hybrid zone

### 4.1 Clinal analyses

Five hundred and twenty individuals from 13 locations were genotyped at one mtDNA SNP and nine microsatellite markers to confirm the previously observed locations of the cline centres for these nuclear and mitochondrial markers [18,20,21,51].

**Figure 2.**
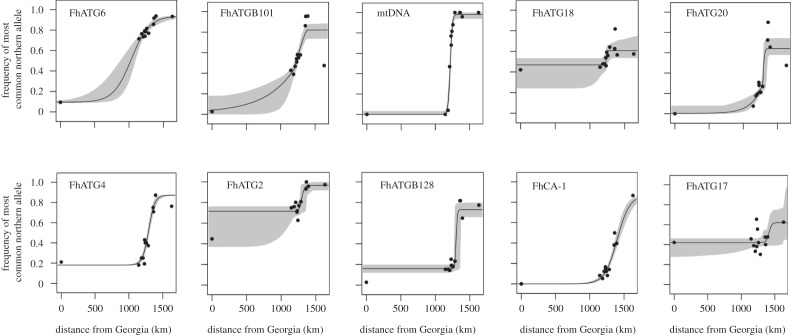
Shape of the cline of nine microsatellite loci and an mtDNA SNP of *F. heteroclitus* populations distributed along the coast of North America as determined using HZAR. Grey shading indicates the 2-unit support envelope around the cline. Distance refers to distance from Georgia as in table 1.

All microsatellite loci and the mtDNA SNP marker exhibited clinal variation along the coast (figure 2; electronic supplementary material, table S3 and figure S1), as has been previously observed [18,20,21,51]. Clines were also fitted independently for each sex and did not differ. The mtDNA cline centre was extremely close to our sampled location no. 4 (Metedeconk Creek). The clines generated by the microsatellite loci FhATGB101 and FhATG18 had their centres within the width of the mitochondrial cline (table 3), while FhATG20, FhATG4, FhATG2 and FhATGB128 had similar cline centres that were located slightly to the north of this range (table 3). Further to the north were the cline centres for the loci FhCA-1 and FhATG17, while the cline centre for the marker FhATG6 was substantially south of the mtDNA cline centre (table 3). The likelihood ratio test showed no significant difference between a model where all clines were constrained to the same centre and width (log-likelihood=−4869.10;parameters=6) and a model where these parameters were allowed to vary independently among loci (log-likelihood=−4645.82; parameters=5; likelihood ratio test: *χ*^2^=0.094, d.f.=6,*p*>0.05; electronic supplementary material, table S4 and figure S4).

**Table 3.** Parameters for mitochondrial and microsatellite allele frequency clines (with 2-unit support limits) as estimated with HZAR. Model selection based on lowest AIC value, as indicated in bold for each locus.

					model AIC			
locus name	centre (km)	width (km)	*p*_min_	*p*_max_	null	I	II	III
FhATG6	1035.57 (872.60,1102.30)	525.77 (347.17, 950.62)	0.09 (fixed)	0.94 (fixed)	104.387	**11.734**	15.031	22.600
FhATGB101	1216.19 (1207.30, 1250.95)	377.07 (257.38, 457.69)	0.01 (0.01, 0.10)	0.96 (0.89, 1.0)	193.265	99.29	75.779	**67.566**
mtDNA	1217.47 (1212.52, 1222.23)	57.89 (46.13, 72.11)	0.00 (0.00, 0.02)	0.98 (0.95, 1.00)	366.149	38.951	**21.727**	29.046
FhATG18	1238.46 (1177.50, 1268.31)	7.23 (1.47, 297.40)	0.47 (0.34, 0.51)	0.61 (0.56, 0.67)	26.395	21.223	**15.922**	24.875
FhATG20	1288.67 (1281.02, 1327.94)	43.59 (8.91, 115.26)	0.003 (0, 0.05)	0.64 (0.59, 0.72)	195.100	68.82	46.03	**37.43**
FhATG4	1291.37 (1277.59, 1306.67)	179.23 (139.44, 229.79)	0.18 (fixed)	0.87 (fixed)	197.64	**24.630**	28.257	34.347
FhATG2	1297.11 (1199.43, 1356.68)	73.24 (0.038, 467.43)	0.72 (0.47, 0.75)	0.96 (0.93, 1.0)	92.462	32.921	**32.855**	32.919
FhATGB128	1301.02 (1285.85, 1356.91)	33.98 (0.004, 99.28)	0.16 (0.13, 0.19)	0.73 (0.67, 0.79)	242.700	39.626	**24.446**	28.374
FhCA-1	1398.94 (1371.88, 1434.90)	363.30 (296.29, 459.45)	0 (fixed)	1.0 (fixed)	164.162	**18.286**	21.263	26.942
FhATG17	1413.30 (1295.85, 1699.83)	80.05 (0.33, 1522.26)	0.42 (0.30, 0.45)	0.62 (0.47, 1.00)	30.973	30.254	**29.783**	38.029

**Figure 3.**
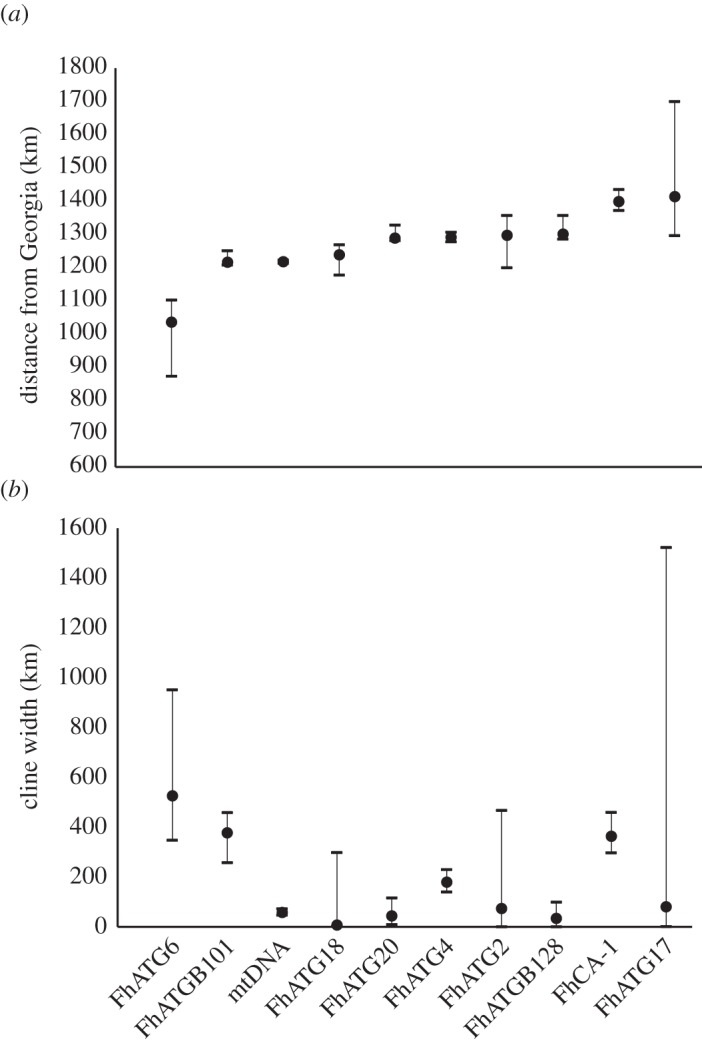
Centre as distance in kilometres from Georgia (*a*) and width (*b*) estimates with 2-unit support limits as estimated using HZAR.

Assuming that the secondary contact of the northern and southern subspecies of *F. heteroclitus* occurred sometime after the beginning of the most recent glacial retreat, approximately 15 000 generations have passed since the secondary contact of these killifish forms. Using a conservative dispersal estimate of 2 km, as derived from mark–recapture data [52], and solving equation (2.1) for *w*, a similarly conservative estimate for the width of the neutral cline is 614 km. Of the estimates for cline width in table 3, only two loci (FhATG6 and FhATG17) have cline widths that are not significantly different from the neutral cline estimate, having upper support limits that greatly exceed the neutral estimate of 614 km. It is notable that the remaining clines all have similarly significantly steep clines and quite similar centres (figure 3).

**Figure 4.**
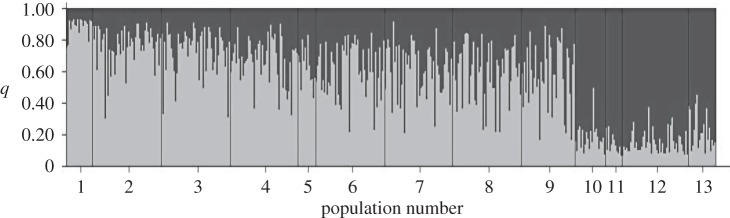
Barplot of admixture values (*q*) from structure, *k*=2. Light grey bars indicate proportion of genome inherited from southern-type parents and black bars indicate proportion of genome inherited from northern-type parents. Population numbering is as in table 1.

Analysis with structure identified the presence of two admixed gene pools as indicated by the elevated Δ*k* calculated using Evanno *et al.*’s method [44] (electronic supplementary material, figure S2). The barplot of *q*-values for *k*=2 (figure 4) clearly shows a transition from pure southern-type individuals dominating the southern-most marsh to an increased proportion of admixed individuals in marsh 2 (RUMFS). Similarly, northern-type individuals predominate in marshes 10–13, with admixed genotypes becoming more common in marshes 9 (Wantagh) and 8 (Cheesequake).

### 4.2 Hybrid index

**Figure 5.**
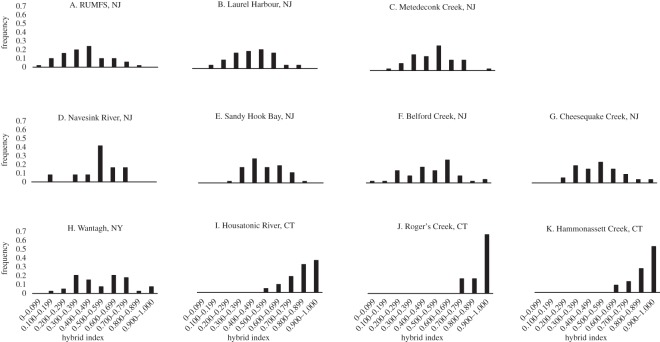
Frequency distributions of hybrid indices as calculated with the program introgress using individuals from Hampton, NH as northern parental types and those from Brunswick, GA as southern parental types. Individuals with a hybrid index of 1.0 are representative of the northern subspecies while those with a hybrid index value of 0.0 identify with the southern subspecies.

Hybrid indices for the multilocus microsatellite data were first calculated for individuals (*n*=432) from 11 populations using individuals from the northernmost (Hampton, NH) and southern-most (Brunswick, GA) sampling locations as parental populations to train the program introgress (figure 5). Consistent with the results of the structure analysis, all populations within the putative hybrid zone were genotypically intermediate between the two extreme populations. Within the putative hybrid zone, the frequency distributions in hybrid index exhibited unimodal or flat patterns (figure 5*a*–*h*) and were progressively more similar to the extreme northern population at sites north of Long Island (figure 5*i*–*k*).

**Figure 6.**
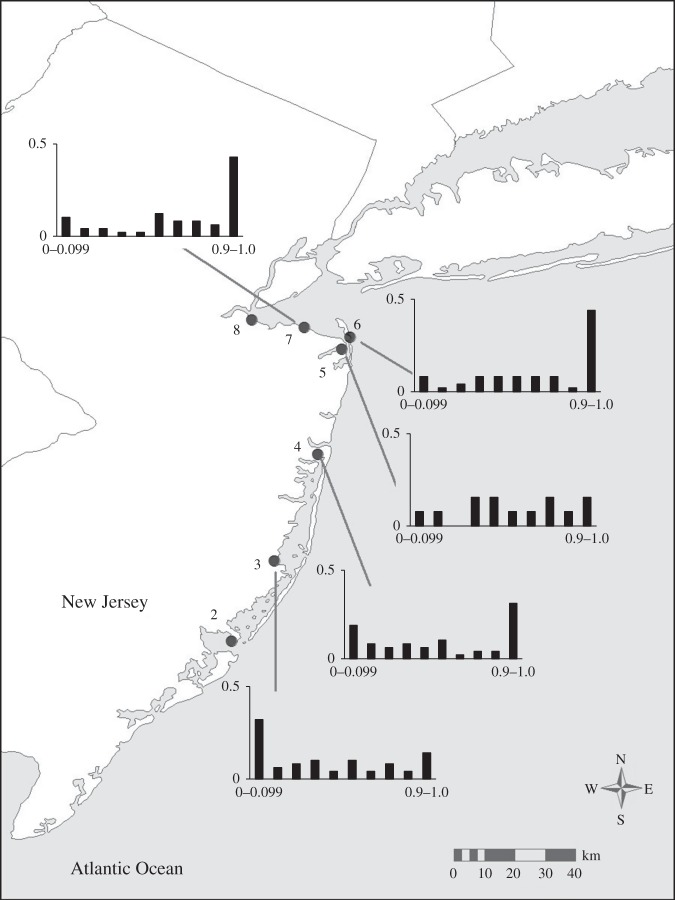
Frequency distributions of hybrid indices calculated using the most likely ‘southern’ and ‘northern’ parents. A hybrid index of 0 represents a pure RUMFS (location 2) genotype while a value of 1 indicates a pure Cheesequake (location 8) genotype.

**Figure 7.**
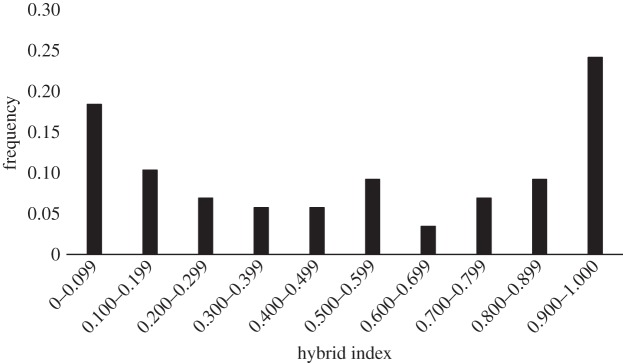
Hybrid index as calculated using introgress for individuals collected in Metedeconk across two years. A hybrid index of 0 represents a pure RUMFS (location 2) genotype while a value of 1 indicates a pure Cheesequake (location 8) genotype.

The individuals collected from the extremes of the species’ distribution represent the pure forms of the northern and southern subspecies of *F. heteroclitus*, but it is unlikely that fish from these locations represent the true parental genotypes of individuals within the hybrid zone, due to their great distance from the zone and the clinal patterns of allele frequencies at the microsatellite loci. Thus, based on the results from the structure analysis (above), individuals from marshes 2 (RUMFS) and 8 (Cheesequake) were chosen to represent the most likely ‘southern’ and ‘northern’ parents, respectively, with which to train introgress for the estimation of hybrid indices for populations of killifish located within the hybrid zone (locations 3–7; *n*=311). The sample from Wantagh, NY (Marsh 9) yielded individual admixture proportions, *q*, and mean-*q* values (figure 4; electronic supplementary material, figure S3) similar to those observed among the samples from Cheesequake and fish from these two populations are not genetically distinct (*F*_ST_=0.00297;*p*=0.17117). However, we elected to use the individuals from Cheesequake as representative of the northern parental types in the hybrid index analysis due to the relative proximity of these individuals to the central populations of interest in this analysis as it is unlikely that these killifish disperse long distances along the coast. There was a gradual transition from a more southern hybrid index in the south of the hybrid zone to a more northern hybrid index towards the northern edge of the zone (figure 6). However, in the central marsh (4, Metedeconk), the microsatellite data show a lower frequency of intermediate genotypes and a higher frequency of pure northern and southern types upon visual inspection. Formal analysis using the R package diptest led to the rejection of the null hypothesis of unimodality (*D*=0.0927,*p*=0.0015) [49]. The frequency distribution of hybrid indices in marsh 4 (Metedeconk) is suggestive of a bimodal pattern. To strengthen this conclusion, we analysed a combined dataset containing an additional 40 individuals sampled at the same location the following year (November 2009). This sample was genetically indistinguishable (*F*_ST_=0.00616,*p*>0.05) from the original samples collected in June 2008. The resulting distribution of hybrid indices generated using this larger sample size (*n*=89) yielded an even more pronounced bimodal pattern (*D*=0.0805, *p*<0.0001; figure 7). Thus, the pattern of hybrid indices observed in Metedeconk cannot be explained by a unimodal distribution, and is best explained by a bimodal distribution. By contrast, a nearby marsh (5, Navesink) had an essentially even distribution of all hybrid indices. However, there was a relatively small sample size from this marsh (*n*=13), so it is difficult to make firm conclusions about the shape of the distribution of hybrid indices based on this location. Owing to its low sample size, Navesink was excluded from subsequent analyses.

To assess whether the bimodal distribution of hybrid indices among individuals collected from the centrally located Metedeconk marsh was an artefact of the choice of parental populations for training introgress, we also calculated hybrid index for Metedeconk using all possible ‘northern’ and ‘southern’ parental combinations from within the hybrid zone (electronic supplementary material, figure S5). All comparisons yielded varying degrees of a bimodal pattern in frequency distribution of hybrid index at this marsh.

## 5. Discussion

In this study, we used a combination of mitochondrial DNA and microsatellite markers and a multilocus analytical approach to characterize genetic variation in killifish, *F. heteroclitus*. We thus extended the previous analyses of Adams *et al.* [20] and Duvernell *et al.* [21], who examined variation at these microsatellites but did not calculate a hybrid index, and those of Gonzáles-Villasenor & Powers [18] and Smith *et al.* [19], who examined mtDNA variation but did not examine variation in the nuclear genome. We confirmed that the location of the centre of the mtDNA cline is in north central New Jersey in agreement with previous studies [18,19]. Analysis of mtDNA and microsatellite cline shapes with Cfit7 suggests that a model constraining all clines to the same centre and width has equal likelihood to a model allowing these parameters to vary among loci. This is in direct contrast to previous analyses of these markers [20], which suggested that nuclear and mtDNA cline centres were substantially discordant. Locus-by-locus examination of our cline centre estimates (from HZAR) suggests that the majority of the microsatellite clines are concordant with the mtDNA cline, based on examination of 2-unit support limits. In addition, the loci that have their centres located closest to the mtDNA cline centre all have widths that are less than that predicted under a neutral model, indicating that these clines may be maintained by the same strength and type of selection. By contrast, the two clines with widths that are not significantly different from a neutral cline width (FhATG6 and FhCA-1) have centres (and associated 2-unit support limits) located outside the borders of the hybrid zone, distant from the mitochondrial cline centre. Thus, amalgamating these markers together into a single nuclear cline may result in a false estimate of the nuclear cline centre. This observation suggests that there is additional complexity to the genetic patterns in this species that has not been fully considered in previous analyses.

In addition, we found evidence of a bimodal distribution of nuclear hybrid indices at a marsh at the mtDNA cline centre. This bimodal pattern was strengthened when additional genetically indistinguishable samples from a subsequent year were added to the analysis and persisted when varying combinations of populations from within and immediately adjacent to the hybrid zone were used as reference parental populations. Together, these data suggest the possibility of reproductive isolating barriers acting against first generation and backcrossed types in populations close to the mtDNA cline centre to maintain this genetic structure. However, this pattern was not evident when populations from the extremes of the species distribution were used as parental populations, indicating that there is substantial gene flow across this hybrid zone at a larger scale.

Bimodal hybrid zones have been described across a wide range of taxa including fish (e.g. [53,54]), molluscs (e.g. [55,56]), insects (e.g. [57,58]), birds (e.g. [41,59]), plants (e.g. [60,61]), reptiles (e.g. [62,63]) and mammals (e.g. [64]). The presence of a bimodal hybrid zone is an important genetic signature of reproductive isolation, in which parental types dominate a particular location, and a deficit of hybrid types is observed. The results of our hybrid index analyses coupled with the observation of elevated occurrences of linkage disequilibrium in these marshes near the centre of the mtDNA and several microsatellite clines reinforce the conclusion that there is some form of reproductive isolation between the northern and southern types of *F. heteroclitus* influencing population structure in this region. However, our results also indicate that this reproductive isolation is not complete, and that there is still substantial gene flow between the subspecies. This conclusion is consistent with the broad clines observed at some loci in *F. heteroclitus* [17,22,65].

The main factors that could promote the formation of the patterns we observed are incomplete premating and/or pre-zygotic isolation, selection against backcross types, and the inability of recombination to breakdown co-adapted gene complexes (as evidenced by the presence of linkage disequilibrium among genetic markers within the hybrid zone [7]). This maintenance of non-random associations of particular genotypic combinations in an area where two subspecies are known to be sympatric can be the result of several phenomena. For example, selection against hybrid offspring produced by matings between ‘more pure’ northern-type and southern-type parents that are constantly migrating in from neighbouring populations can create a balance between dispersal and selection [9]. Alternatively, phenomena such as positive assortative mating can also produce these genetic associations and distributions of hybrid indices in the hybrid zone [7]. Indeed, these phenomena are not mutually exclusive, and both may be operating. For example, strong selection against hybrids harbouring maladapted combinations of genotypes as the result of matings between genetically distinct parental types is likely to result in reinforcement through the strengthening of pre-zygotic barriers (i.e. assortative mating, ecological character displacement) [66]. Such pre-zygotic barriers tend to be favoured in response to existing post-zygotic barriers (i.e. hybrid sterility, hybrid inviability) because they operate early in an organism’s life cycle and as such will reduce an individual’s incurred cost of wasting gametes in production of less fit hybrid offspring [67]. At this point, it is reasonable to conclude that killifish residing in the hybrid zone are experiencing some combination of pre-zygotic and/or post-zygotic isolation that results in reduced numbers of F_1_ and backcross genotypes in the population. It is important to note that at this time we are unable to distinguish whether a pre-zygotic or post-zygotic barrier is the main contributor to the reproductive isolation we are seeing among these subspecies, because the minimum size of fish retained by the minnow trap was 32 mm. Thus, we cannot differentiate among a scenario in which the hybrids are not being produced at all (pre-zygotic isolation) or one in which the hybrids are being produced but are not surviving beyond this minimum size (post-zygotic isolation).

## 6. Conclusion

In summary, we have found evidence of a bimodal hybrid zone connecting the northern and southern subspecies of the killifish, *F. heteroclitus*, for multilocus genotypes assessed with highly variable microsatellite markers. This bimodal pattern in the centrally located marshes near the centres of the steep mtDNA D-loop and microsatellite clines suggests some level of pre- and/or post-zygotic reproductive isolation is operating to prevent the complete merging of these two subspecies [7]. Together, these results emphasize the importance of collecting data on both the nuclear and cytoplasmic genomes and considering multilocus genotypes when trying to uncover the underlying genetic structure of closely related hybridizing taxa.

## Supplementary Material

Supplemental Figures and Tables PDF file containing figures S1-S5, and tables S1, S3 and S4.

## Supplementary Material

S2 Table Raw Microsatellites Excel file containing all raw data analyzed in this paper.

## Data accessibility

Multilocus microsatellite genotypes of all individuals included in this study are available in the electronic supplementary material.
