# Supplementary material for: Evidence for a bimodal distribution of hybrid indices in a hybrid zone with high admixture
Source: R Soc Open Sci. 2015 Dec 9;2(12):150285. doi: 10.1098/rsos.150285 (PMC4807441; doi:10.1098/rsos.150285)
Supplement: Supplemental Figures and Tables PDF file containing figures S1-S5, and tables S1, S3 and S4. [file rsos150285supp1.pdf]

S1 Figure. Frequency of each allele present (y-axis) in each population (y-axis) for each locus.

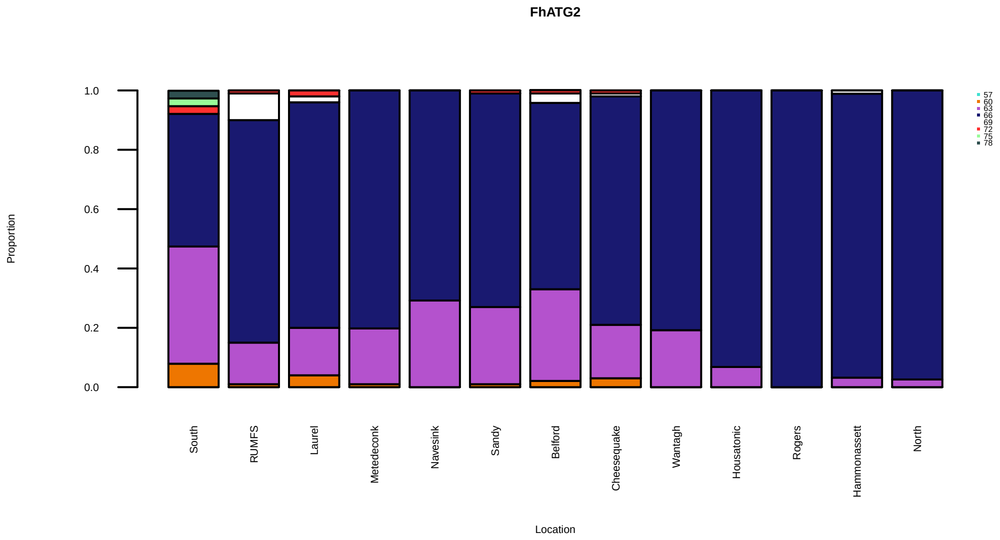
FhATG2
Proportion
1.0
0.8
0.6
0.4
0.2
0.0
South
RUMFS
Laurel
Metedeconk
Navesink
Sandy
Belford
Cheesequake
Wantagh
Housatonic
Rogers
Hammonassett
North
Location
57
60
63
66
69
72
75
78

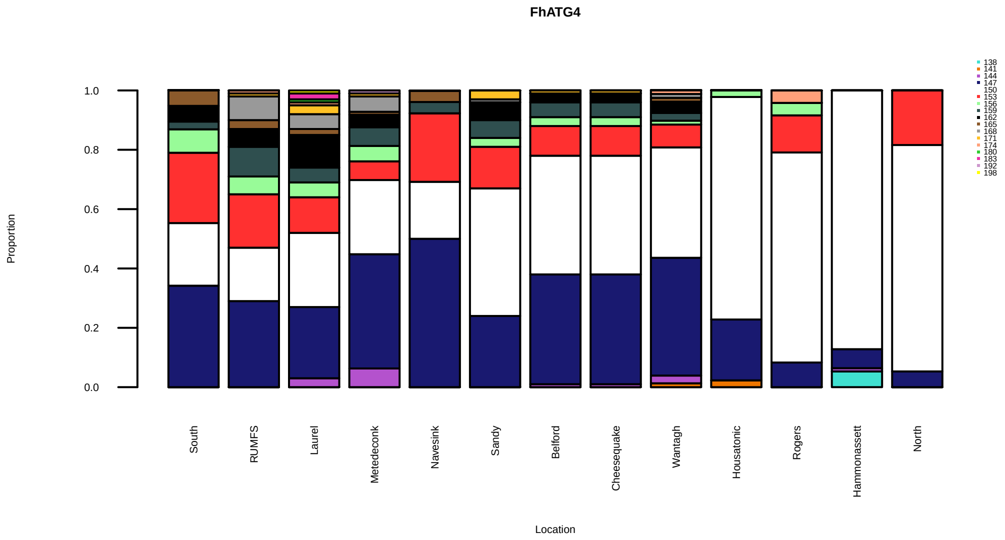
FhATG4
Proportion
1.0
0.8
0.6
0.4
0.2
0.0
138
141
144
147
150
153
156
159
162
165
168
171
174
180
183
192
198
South
RUMFS
Laurel
Metedeconk
Navesink
Sandy
Belford
Cheesequake
Wantagh
Housatonic
Rogers
Hammonassett
North
Location

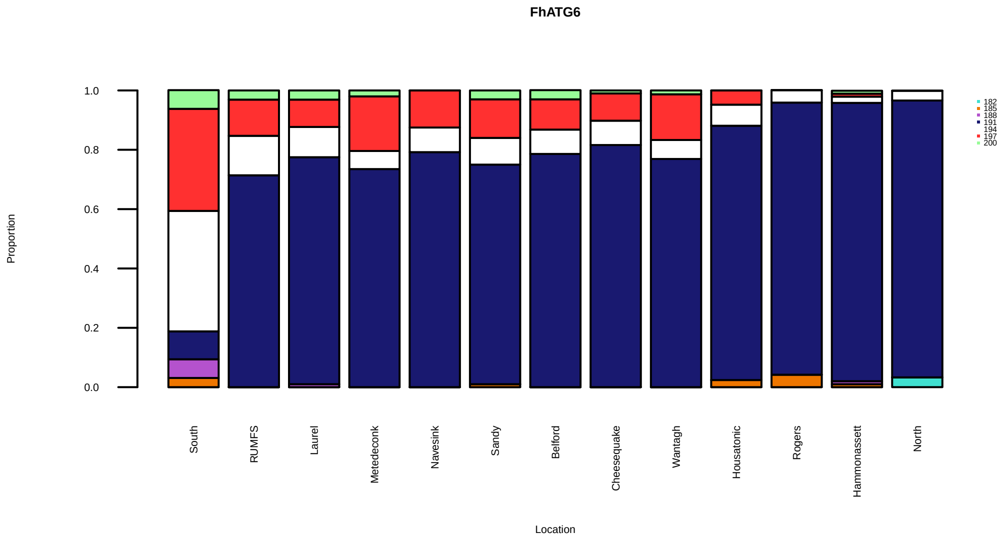
FhATG6
Proportion
1.0
0.8
0.6
0.4
0.2
0.0
South
RUMFS
Laurel
Metedeconk
Navesink
Sandy
Belford
Cheesequake
Wantagh
Housatonic
Rogers
Hammonassett
North
Location
182
185
188
191
194
197
200

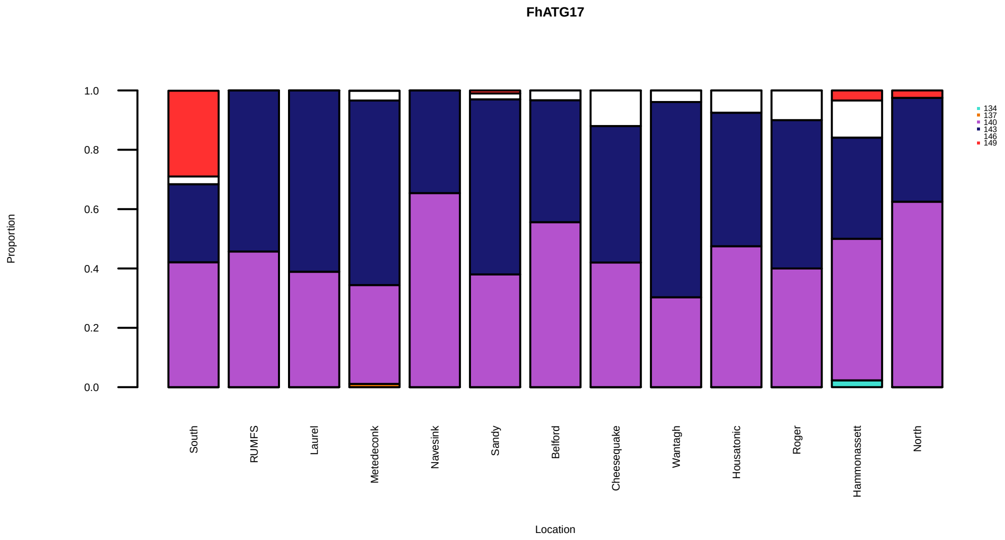

FhATG17
Proportion
1.0
0.8
0.6
0.4
0.2
0.0
South
RUMFS
Laurel
Metedeconk
Navesink
Sandy
Belford
Cheesequake
Wantagh
Housatonic
Roger
Hammonassett
North
Location
134
137
140
143
146
149

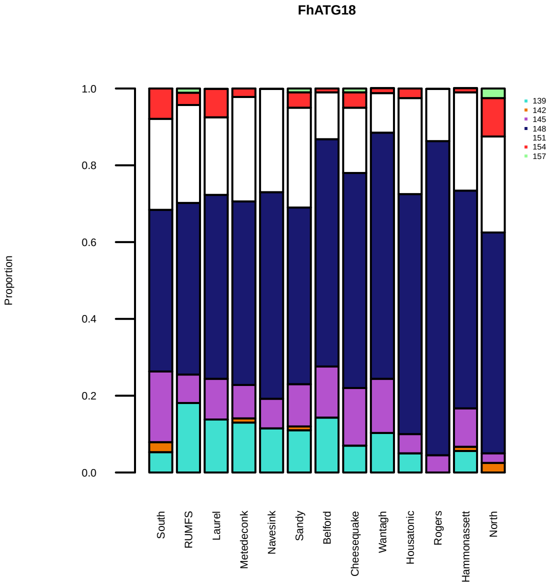
FhATG18
Proportion
0.0
0.2
0.4
0.6
0.8
1.0
139
142
145
148
151
154
157
South
RUMFS
Laurel
Metedeconk
Navesink
Sandy
Belford
Cheesequake
Wantagh
Housatonic
Rogers
Hammonassett
North

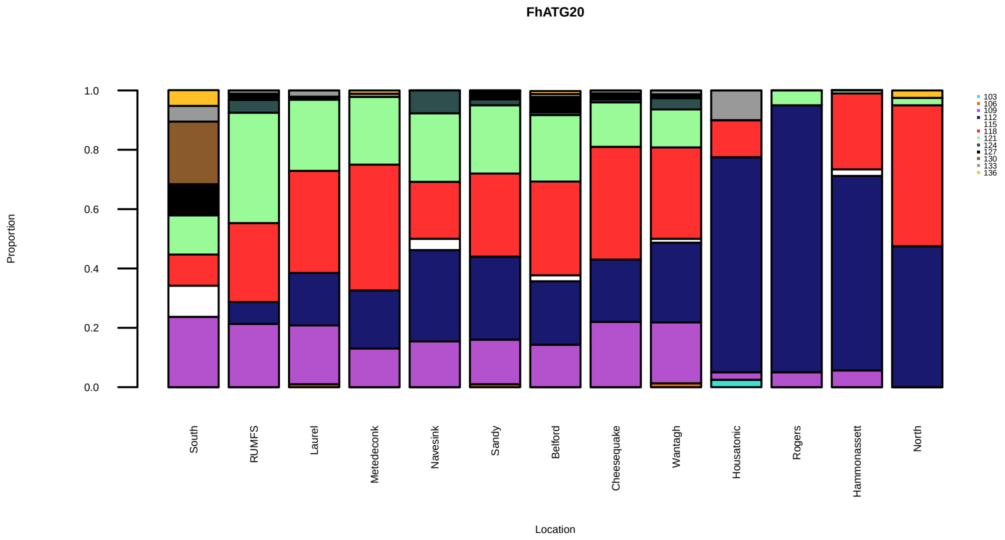
FhATG20
Proportion
0.0
0.2
0.4
0.6
0.8
1.0
South
RUMFS
Laurel
Metedeconk
Navesink
Sandy
Belford
Cheesequake
Wantagh
Housatonic
Rogers
Hammonassett
North
Location
103
106
109
112
115
118
121
124
127
130
133
136

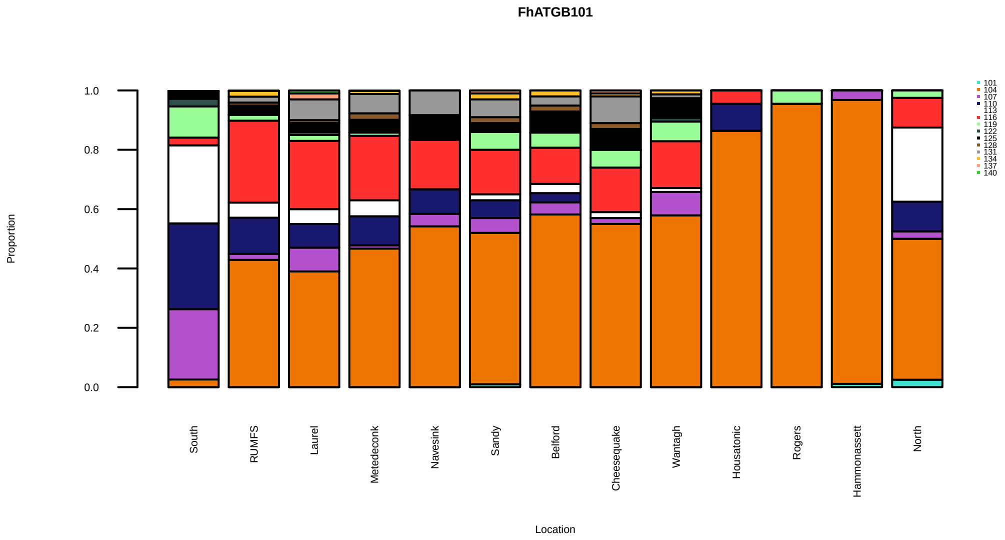
FhATGB101
Proportion
1.0
0.8
0.6
0.4
0.2
0.0
South
RUMFS
Laurel
Metedeconk
Navesink
Sandy
Belford
Cheesequake
Wantagh
Housatonic
Rogers
Hammonassett
North
Location
101
104
107
110
113
116
119
122
125
128
131
134
137
140

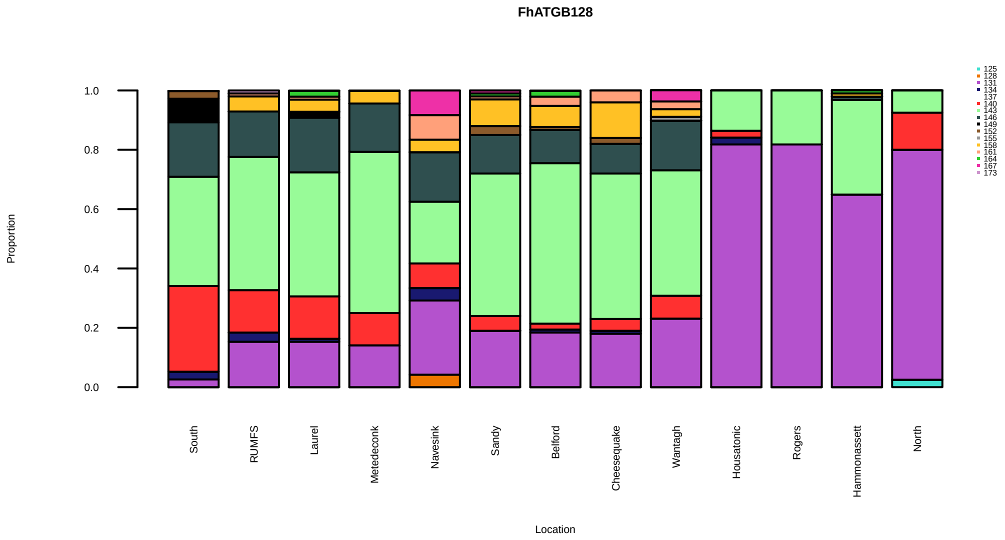

FhATGB128
Proportion
1.0
0.8
0.6
0.4
0.2
0.0
South
RUMFS
Laurel
Metedeconk
Navesink
Sandy
Belford
Cheesequake
Wantagh
Housatonic
Rogers
Hammonassett
North
Location
125
128
131
134
137
140
143
146
149
152
155
158
161
164
167
173

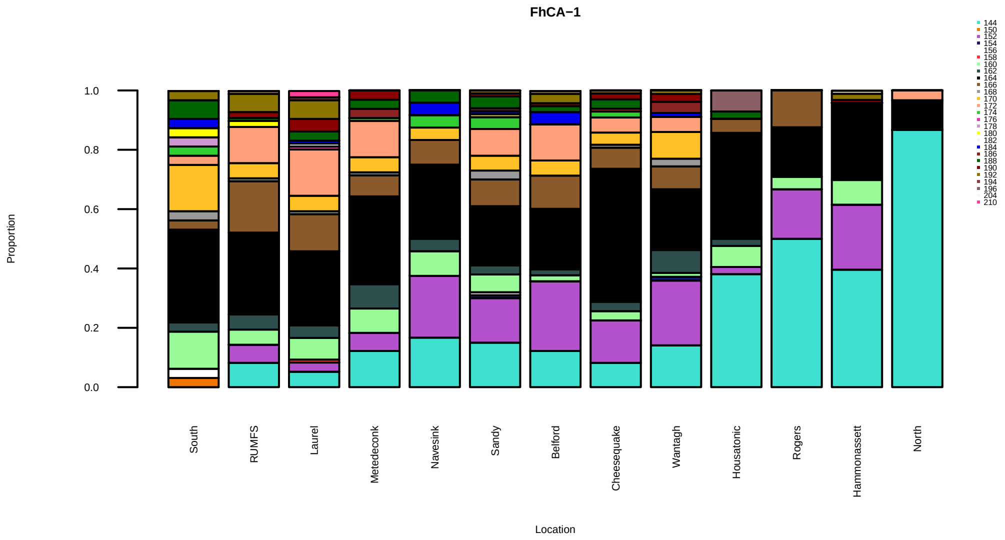
FhCA−1
Proportion
1.0
0.8
0.6
0.4
0.2
0.0
South
RUMFS
Laurel
Metedeconk
Navesink
Sandy
Belford
Cheesequake
Wantagh
Housatonic
Rogers
Hammonassett
North
Location
144
150
152
154
156
158
160
162
164
166
168
170
172
174
176
178
180
182
184
186
188
190
192
194
196
204
210

S2 Figure. Harvester analysis of *structure* output showing support for k=2 clusters.

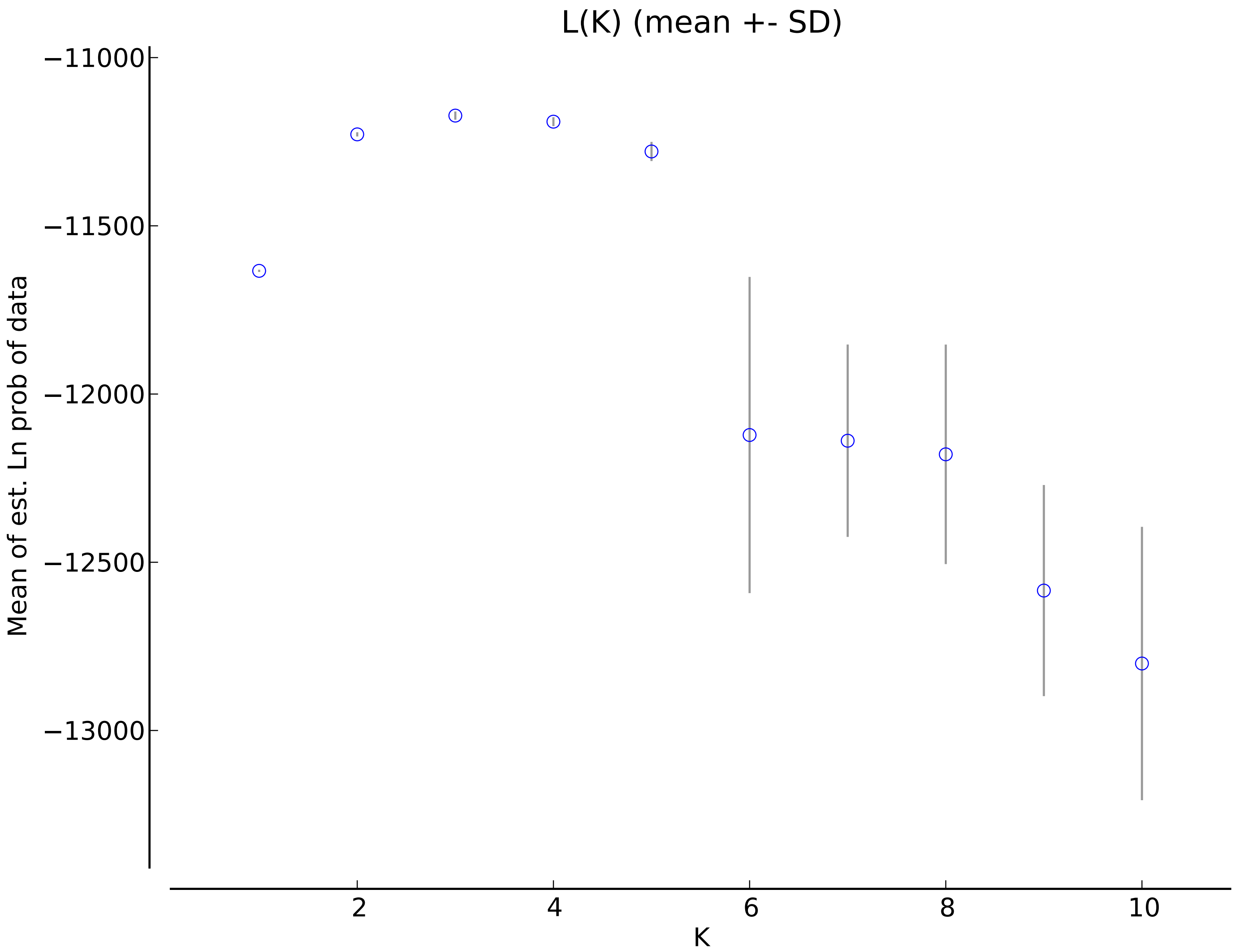
L(K) (mean +- SD)
−11000
−11500
−12000
−12500
−13000
Mean of est. Ln prob of data
2
4
6
8
10
K

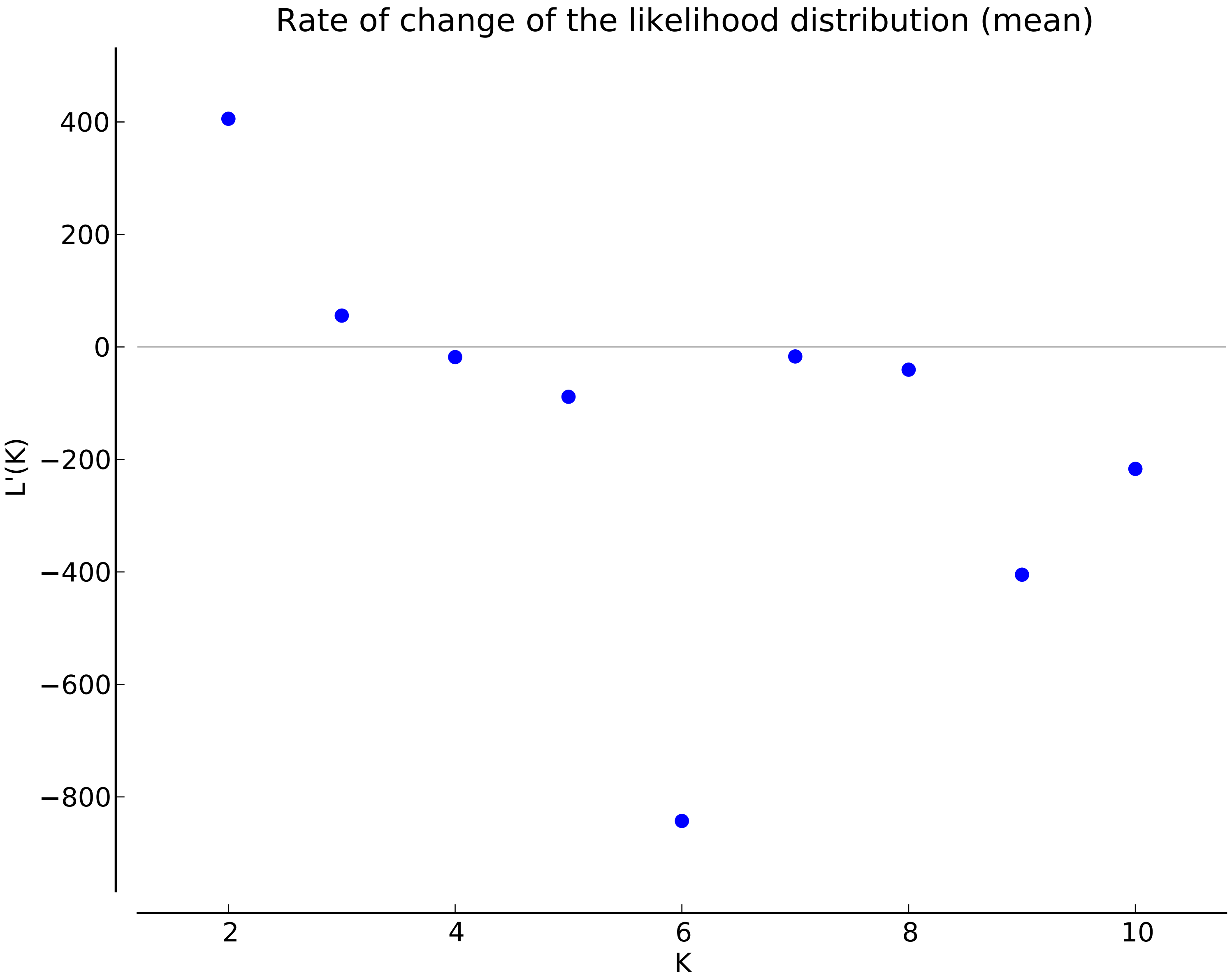

Rate of change of the likelihood distribution (mean)
400
200
0
−200
−400
−600
−800
L'(K)
2
4
6
8
10
K

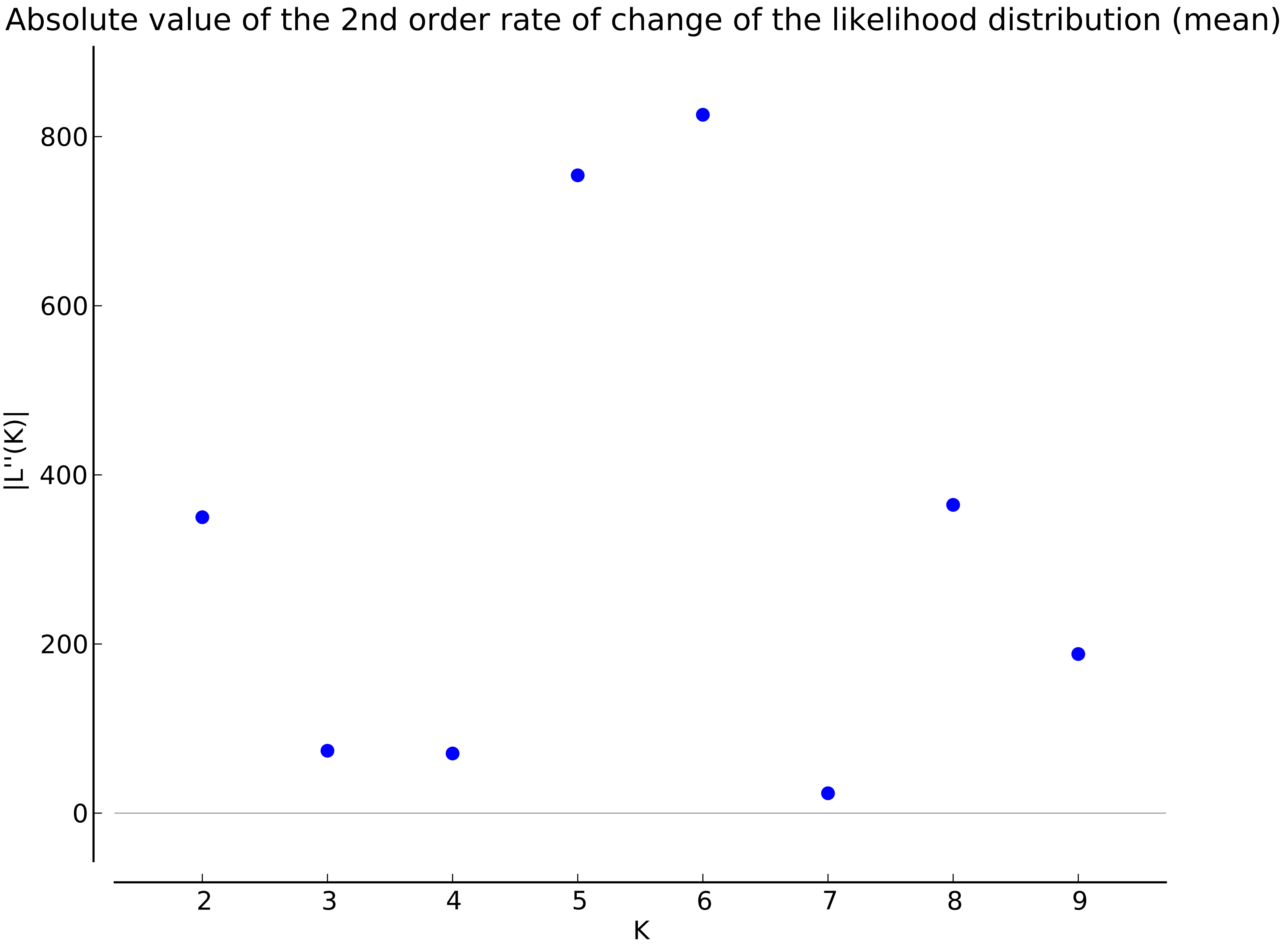

Absolute value of the 2nd order rate of change of the likelihood distribution (mean)
|L''(K)|
0
200
400
600
800
2
3
4
5
6
7
8
9
K

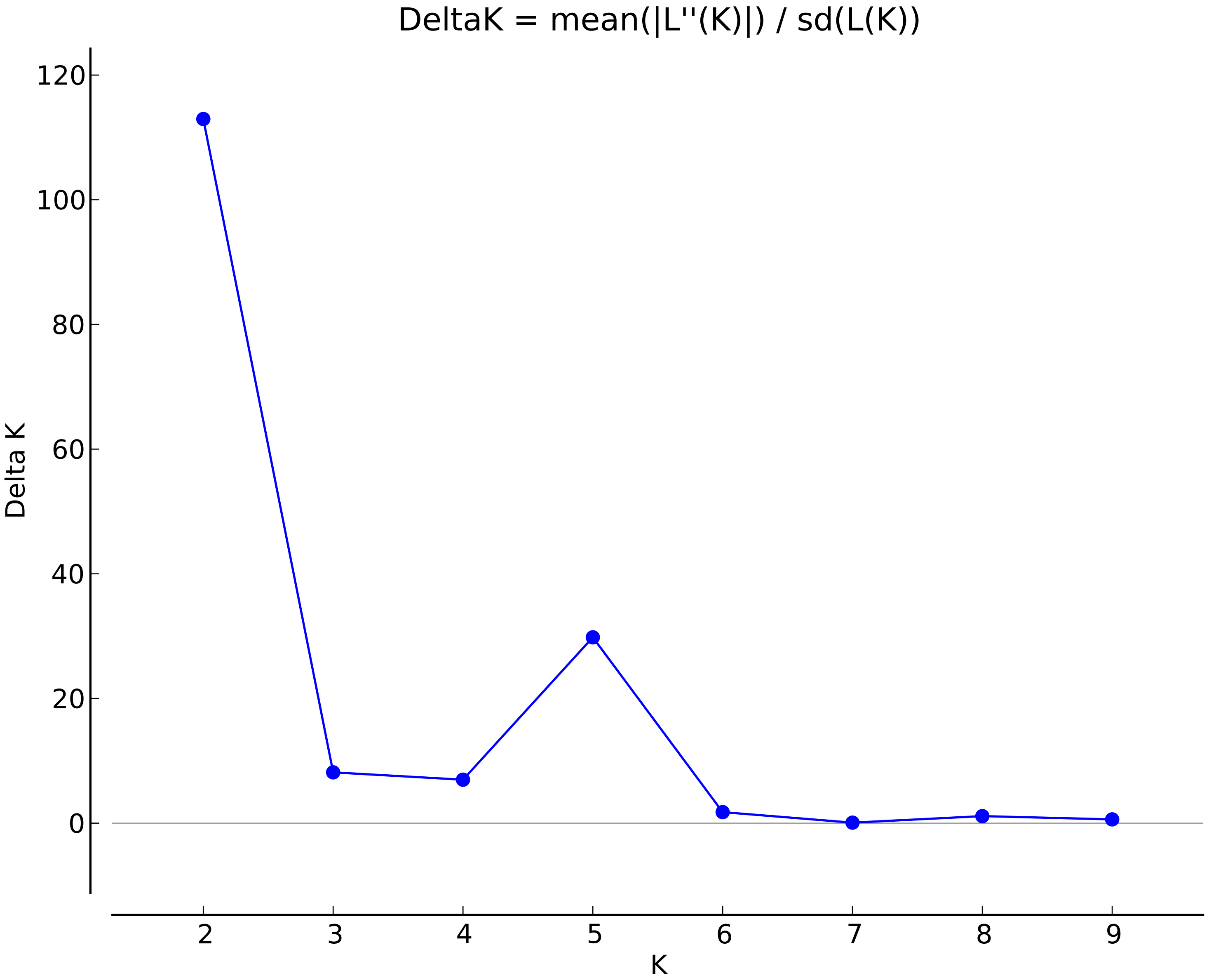
DeltaK = mean(|L''(K)|) / sd(L(K))
Delta K
120
100
80
60
40
20
0
2
3
4
5
6
7
8
9
K

Evanno table

# K	Reps	Mean LnP(K)	Stdev LnP(K)	Ln'(K)	|Ln''(K)|	Delta K
1	10	-11633.83	0.116	NA	NA	NA
2	10	-11228.15	3.0981	405.68	349.94	112.952455
3	10	-11172.41	9.0527	55.74	73.77	8.148914
4	10	-11190.44	10.1167	-18.03	70.53	6.971658
5	10	-11279	25.3007	-88.56	754.23	29.81066
6	10	-12121.79	466.7155	-842.79	825.85	1.769493
7	10	-12138.73	282.7365	-16.94	23.49	0.083081
8	10	-12179.16	323.0629	-40.43	364.53	1.128356
9	10	-12584.12	310.5894	-404.96	188.15	0.605784
10	10	-12800.93	403.1152	-216.81	NA	NA

Evanno table

# K	Reps	Mean LnP(K)	Stdev LnP(K)	Ln'(K)	|Ln''(K)|	Delta K
1	10	-11633.83	0.116	NA	NA	NA
2	10	-11228.15	3.0981	405.68	349.94	112.952455
3	10	-11172.41	9.0527	55.74	73.77	8.148914
4	10	-11190.44	10.1167	-18.03	70.53	6.971658
5	10	-11279	25.3007	-88.56	754.23	29.81066
6	10	-12121.79	466.7155	-842.79	825.85	1.769493
7	10	-12138.73	282.7365	-16.94	23.49	0.083081
8	10	-12179.16	323.0629	-40.43	364.53	1.128356
9	10	-12584.12	310.5894	-404.96	188.15	0.605784
10	10	-12800.93	403.1152	-216.81	NA	NA

S3 Figure. The mean (q-bar) and 95% confidence interval of q values for each coastal killifish population as calculated for k=2, and each population's distance from Georgia (x-axis) as in Table 1.

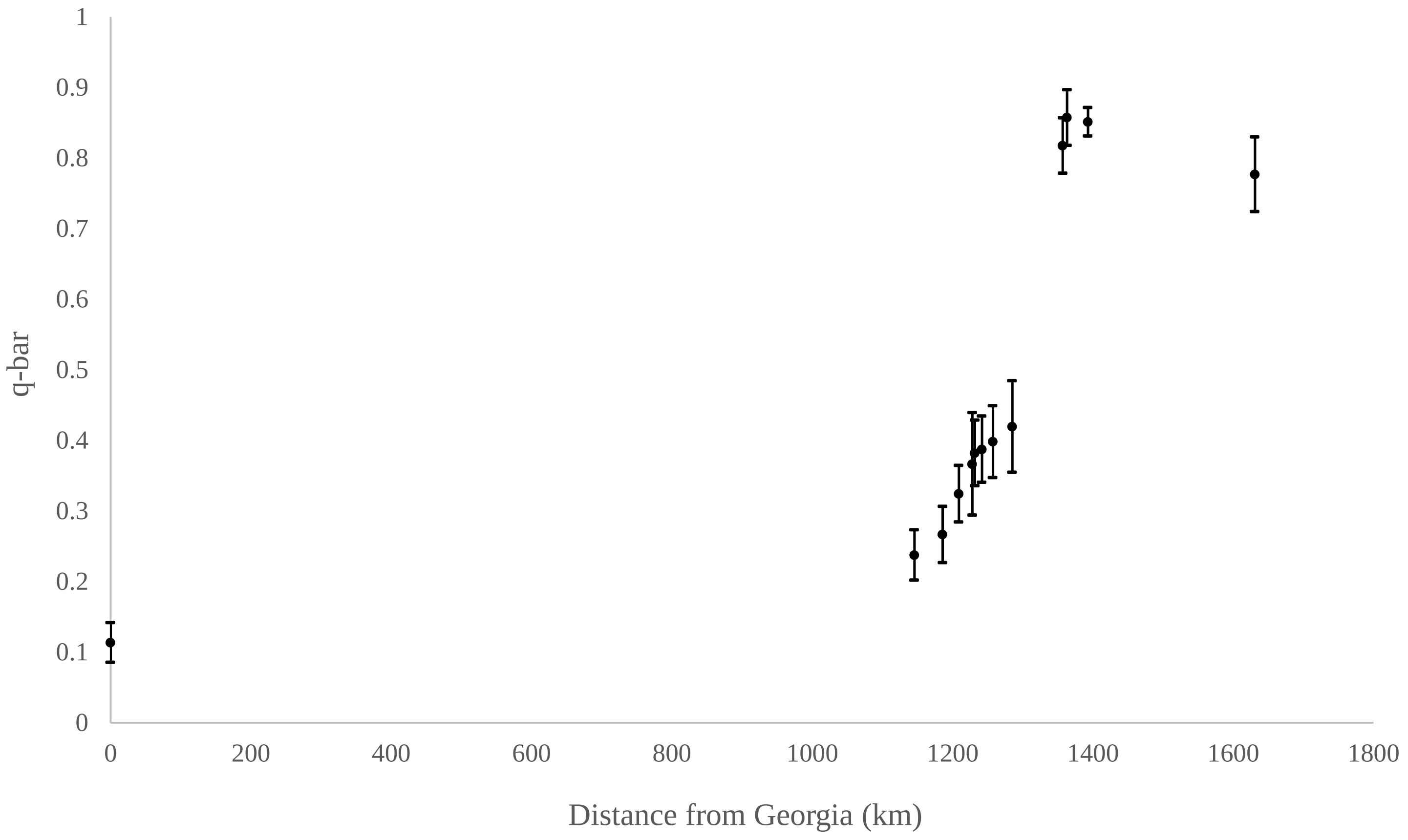

S4 Figure. *Cfit7* best fit clines as estimated using the frequency of the most common allele in the northernmost sampling location for each locus in this study: A) FhATG6, B) FhATGB101, C) mtDNA, D) FhATG18, E) FhATG20, F) FhATG4, G) FhATG2, H) FhATGB128, I) FhCA-1, J) FhATG17. Red squares represent the cline centres and blue triangles delimit the widths of the clines.

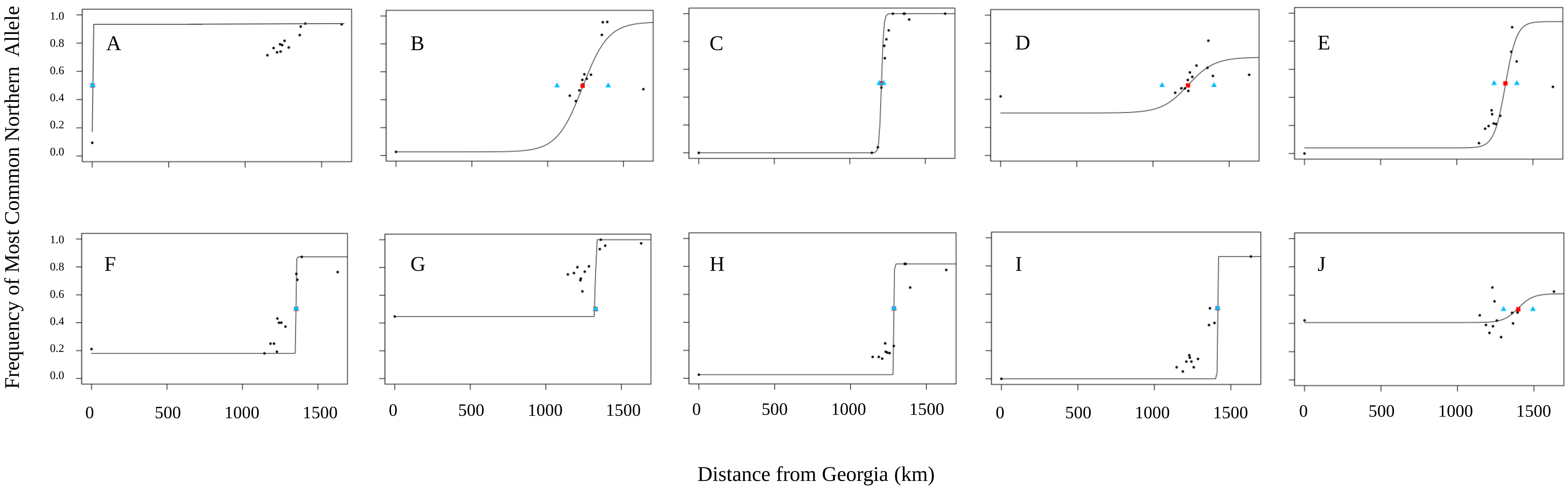

S5 Figure. Frequency distributions in hybrid index for individuals from Metedeconk Creek as calculated using various populations from within and proximate to the killifish hybrid zone as parental references. “Southern” parental populations are indicated with red font, while “northern” parental populations are indicated with blue font. Individuals with a hybrid index approaching 0 are genetically more similar to the “southern” parent population, while those with a hybrid index approaching 1 identify more closely with the “northern” parent population.

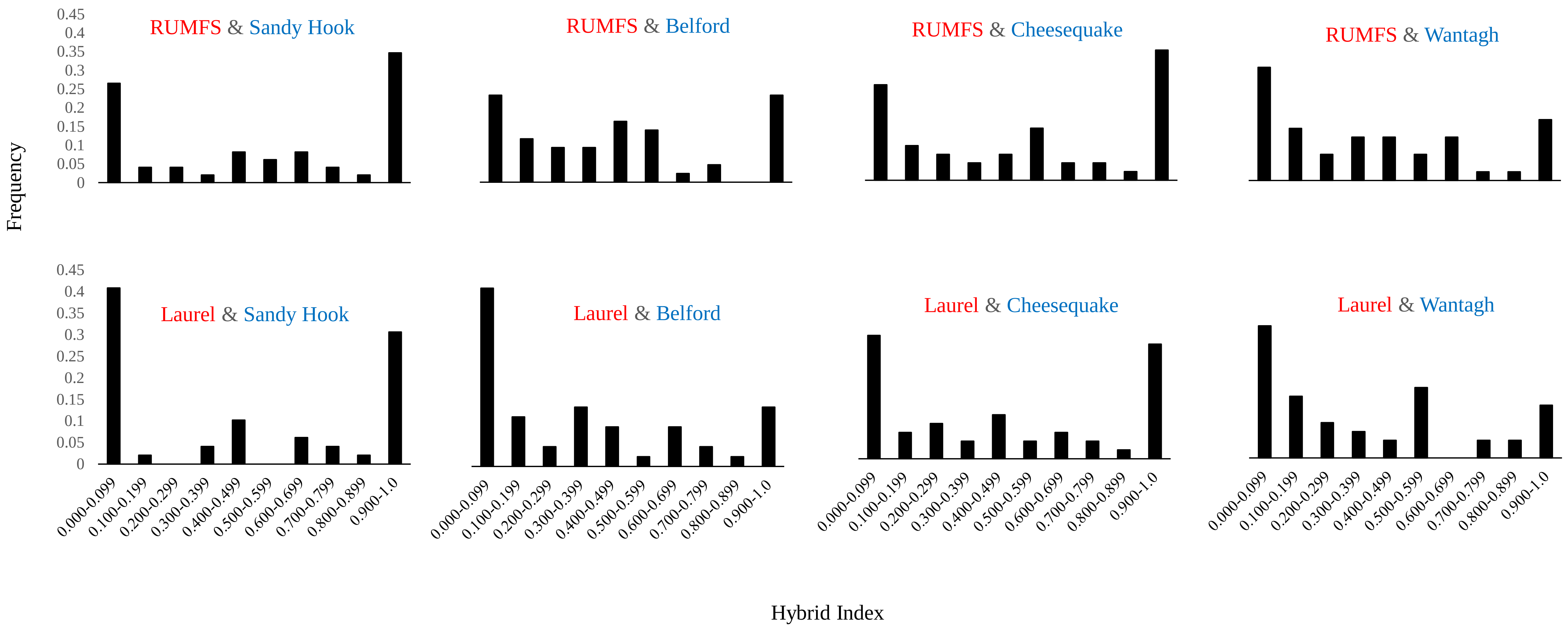

S1 Table. PCR conditions for microsatellite loci. Values are temperature and duration, respectively. Each reaction began with initial denaturation of 95°C for 2 minutes and ended with a final extension of 72°C for 5 minutes.

Locus	Denaturation 1	Annealing 1	Extension 1	Denaturation 2	Annealing 2	Extension 2
FhCA-1	94°, 30s	52°, 45s	72°, 1 min	94°, 30	56°, 45s	72°, 1 min
FhATG-6	94°, 30s	63°, 45s	72°, 1 min	NA	NA	NA
FhATG-2 FhATG-4 FhATG-B101 FhATG-B128	94°, 30s	58°, 45s	72°, 1 min	NA	NA	NA
FhATG-17 FhATG-18 FhATG-20	94°, 30s	54°, 45s	72°, 1 min	94°, 30s	59°, 45s	72°, 1 min

S3 Table. Frequency of the northern mtDNA genotype and the most common northern allele for each microsatellite in *F. heteroclitus*. Location numbers correspond to those given in Table 1. Distance from Georgia is calculated as the straight line distance along the coastline. The total number of alleles at each locus and at each location are given in parentheses.

**Location**	**Distance from Georgia (km)**	**mtDNA^{N}**	**FhATG18 (7)**	**FhATG20 (12)**	**FhATG17 (6)**	**FhATG B101 (14)**	**FhATG B128 (16)**	**FhATG2 (8)**	**FhATG4 (17)**	**FhATG6 (7)**	**FhCA-1 (27)**
**1.**	0	0.000	0.421 (6)	0 (8)	0.421 (4)	0.026 (8)	0.026 (7)	0.447 (6)	0.211 (7)	0.094 (6)	0 (15)
**2.**	1145.77	0.000	0.420 (6)	0.070 (7)	0.430 (2)	0.420 (10)	0.150 (8)	0.750 (5)	0.180 (10)	0.714 (4)	0.082 (14)
**3.**	1186.03	0.040	0.450 (5)	0.170 (7)	0.350 (2)	0.390 (12)	0.150 (9)	0.760 (5)	0.250 (14)	0.765 (5)	0.052 (18)
**4.**	1208.94	0.469	0.449 (8)	0.176 (9)	0.294 (6)	0.427 (13)	0.136 (9)	0.741 (4)	0.268 (12)	0.735 (4)	0.122 (13)
**5.**	1228.41	0.769	0.602 (5)	0.258 (8)	0.444 (3)	0.563 (11)	0.227 (11)	0.713 (3)	0.285 (8)	0.792 (3)	0.167 (10)
**6.**	1231.88	0.680	0.460 (7)	0.280 (7)	0.380 (4)	0.510 (12)	0.190 (9)	0.720 (4)	0.430 (8)	0.740 (5)	0.150 (18)
**7.**	1242.06	0.816	0.592 (5)	0.214 (9)	0.543 (3)	0.582 (10)	0.184 (9)	0.615 (5)	0.406 (9)	0.786 (4)	0.122 (13)
**8.**	1257.54	0.880	0.555 (6)	0.233 (8)	0.438 (3)	0.556 (11)	0.169 (9)	0.753 (5)	0.432 (9)	0.816 (4)	0.082 (14)
**9.**	1285.31	1.000	0.641 (5)	0.269 (9)	0.303 (3)	0.579 (9)	0.231 (8)	0.808 (2)	0.372 (11)	0.769 (4)	0.141 (14)
**10.**	1357.23	1.000	0.625 (5)	0.725 (5)	0.475 (3)	0.864 (3)	0.818 (4)	0.932 (2)	0.750 (4)	0.857 (4)	0.381 (8)
**11.**	1363.41	1.000	0.818 (3)	0.900 (3)	0.400 (3)	0.955 (2)	0.818 (2)	1.000 (1)	0.708 (5)	0.917 (3)	0.500 (5)
**12.**	1393.09	0.958	0.548 (7)	0.639 (5)	0.541 (5)	0.962 (3)	0.652 (4)	0.970 (3)	0.873 (5)	0.938 (6)	0.396 (7)
**13.**	1631	1.000	0.575 (6)	0.475 (4)	0.625 (3)	0.475 (7)	0.775 (4)	0.974 (2)	0.763 (3)	0.933 (3)	0.867 (3)

S4 Table. Parameters for mitochondrial and microsatellite allele frequency clines as estimated with *Cfit*.

Locus Name	Centre (km)	Slope	Width (km)
FhATG6	0.230	9.8745	4.05
FhATGB101	1230.45	0.1185	337.55
mtDNA	1209.53	1.3600	29.41
FhATG18	1229.88	0.1172	341.30
FhATG20	1319.40	0.2679	149.31
FhATG4	1356.13	9.9985	4.00
FhATG2	1329.58	10.000	4.00
FhATGB128	1286.66	8.9057	4.49
FhCA-1	1412.96	9.9999	4.00
FhATG17	1397.45	0.2088	191.57
